# Screening asymptomatic patients with diabetes for unknown coronary artery disease: Does it reduce risk? An open-label randomized trial comparing a strategy based on exercise testing aimed at revascularization with management based on pharmacological/behavioural treatment of traditional risk factors. DADDY-D Trial (*Does coronary Atherosclerosis Deserve to be Diagnosed and treated early in Diabetics?*)

**DOI:** 10.1186/1745-6215-10-119

**Published:** 2009-12-23

**Authors:** Fabrizio Turrini, Roberto Messora, Paolo Giovanardi, Stefano Tondi, Paolo Magnavacchi, Rita Cavani, Giandomenico Tosoni, Carlo Cappelli, Elisa Pellegrini, Stefania Romano, Augusto Baldini, Romeo Giulietto Zennaro, Marco Bondi

**Affiliations:** 1 Azienda USL di Modena, Medicina Cardiovascolare, Nuovo Ospedale Sant Agostino Estense, 41100 Modena, Italy; 2 Azienda USL di Modena, Cardiologia, Nuovo Ospedale Sant Agostino Estense, 41100 Modena, Italy; 3 Azienda USL di Modena, Servizio di diabetologia, Ospedale Estense, 41100 Modena, Italy

## Abstract

**Background:**

Coronary artery disease is the leading cause of morbidity and mortality in patients with type 2 diabetes. Screening for asymptomatic coronary artery disease with treatment by means of revascularization seems to be an appealing option for prevention. The utility of such a strategy has never been challenged in a randomized trial.

**Methods/Design:**

In the present study a cohort of diabetic patients without any symptoms and without known coronary artery disease will be screened at two diabetes outpatients services. Those with intermediate or high risk (equal or greater than 10% according to the Italian risk chart) will be asked to participate and enrolled. They will be seen and followed in order to provide the best adherence to medical therapy. Half of the patients will be randomized to undergo an exercise tolerance testing while the other group will continue to be regularly seen at diabetes outpatients services. Best medical/behavioral therapy will be offered to both groups. Those patients with a positive exercise tolerance testing will be studied by coronary angiography and treated according to the severity of coronary lesions by percutaneous stenting or surgery.

The objective of the study is to evaluate the efficacy of the screening strategy aimed at revascularization. A cost-effectiveness analysis will be performed at the end of the follow up.

**Discussion:**

The study will provide useful information about prevention and treatment of diabetic patients at high risk of coronary events. It will be made clearer if detection of silent coronary artery disease has to be recommended and followed by treatment. Given the simplicity of the study protocol, it will be easily transferable to the *real world*.

**Trial registration:**

(ClinicalTrials.gov): NCT00547872

## Background

The association between diabetes and cardiovascular disease is well established. Prevalence of cardiac ischemic disease in the general population is between 2 and 4%, while it reaches 55% among adult diabetic patients [1].

Coronary artery disease is the leading cause of death in diabetic patients accounting for more than 70% of the deaths [2,3].

It is well known that patients with type 2 diabetes without prior myocardial infarction have a similar risk of death from coronary artery disease as patients without diabetes with prior myocardial infarction. Diabetes is considered to be a *risk equivalent *of coronary artery disease for future myocardial infarction and cardiovascular death [4].

Given these strong associations, there is clear need to identify patients with type 2 diabetes who are at risk of cardiovascular events before the onset of symptoms, providing that an effective treatment is available [5].

Observational studies reported a higher rate of acute coronary syndromes in diabetic patients with documented but asymptomatic ischemic coronary artery disease [6]. Nevertheless the hypothesis that revascularization will reduce this risk has never been challenged in a randomized trial.

Screening for coronary artery disease in asymptomatic diabetic patients is thus an appealing but still unexplored concept [7].

According to these observations a screening test should be aimed at detection of severe blockages of the coronary arteries that might warrant treatment.

An ideal test suitable for screening should be safe, sensitive, cheap, repeatable and widely available [8]. Exercise tolerance testing seems close to these ideal characteristics although its sensitivity is low compared with new diagnostic tools available today. On the other hand it does not deliver radiations, it is cheap and really widely available [9].

Recommendations about the use of exercise testing as a screening tool are vague because no clear evidence exists. The American College of Cardiology acknowledges the possible value of exercise testing in people with diabetes who are contemplating an exercise program with a strength of recommendation IIa [10]. The American Diabetes Association suggests exercise testing to diabetic patients when two other major cardiovascular risk factors are present (strength of recommendation IIb) [11]. What to do in case of a positive test it is not known.

U.S. Preventive Task Force states that an "adequately powered randomized trial of screening exercise tolerance testing compared with management based on traditional risk factors would *greatly *inform clinical decision making" [12].

## Method/Design

### General objective

The aim of the study is to evaluate efficacy of screening and treatment of asymptomatic coronary disease in diabetic patients at high risk. Screening is achieved by exercise tolerance testing aimed at revascularization, surgical or percutaneous.

### Primary objective

To assess the effectiveness of screening and treatment of coronary artery disease on the occurrence of cardiac events (acute coronary syndromes or cardiac death) in a group of patients with type 2 diabetes considered at intermediate/high risk according to the risk chart.

### Secondary objective

We will also consider and analyse the impact of screening and treatment of coronary artery disease on the occurrence of symptomatic heart failure. Actually diabetic patients run a higher risk of developing symptomatic heart failure especially after an acute coronary syndrome [13].

### Study Design

The aim of the study is to assess whether screening and treatment of asymptomatic coronary artery disease reduces risk of cardiac events and cardiac death. A prospective *open-label *randomized controlled trial comparing a strategy based on exercise testing aimed at revascularization with management based on pharmacological/behavioural treatment of traditional risk factors has been adopted.

**Figure 1:**
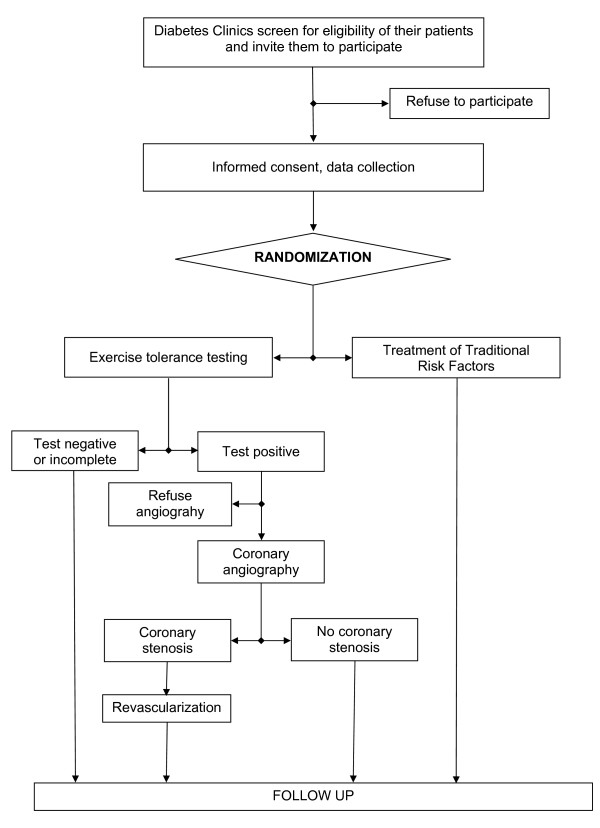
**DADDY-D Trial Flow Chart**.

Eligible patients will be randomly assigned (ratio 1:1) to undergo exercise tolerance testing or to continue to be followed by our diabetes outpatients services. Best medical/behavioral therapy will be offered to both groups. Those patients with a positive exercise tolerance testing will be studied by coronary angiography and treated according to the severity of coronary lesions by percutaneous stenting or surgery (Figure 1).

### Study population

Male and female subjects aged ≥ 50 and < 70 years will be eligible for the study if they meet the following inclusion criteria:

#### Inclusion Criteria

• Clinical diagnosis of type 2 diabetes lasting more than one year, based on standard diagnostic criteria (patients on treatment with oral hypoglycaemic agents or insulin or with fasting plasma glucose > 126 mg/dl on two occasions) irrespective of diabetes treatment

• Cardiovascular risk equal or higher than 10% according to the Italian risk chart available on Progetto Cuore website [14,15]

• Ability to exercise and willingness to comply with all study requirements

#### Exclusion Criteria

• Typical symptoms or known coronary artery disease

• Known or symptomatic heart failure

• Exercise testing or any other stress testing performed in the last year before the enrolment

• Electrocardiogram showing left bundle brunch block or ST depression greater than 1 mm at baseline

• Contraindications to double antiplatelet agents treatment (history of aspirin allergy, bleeding tendency, gastrointestinal haemorrhage or peptic ulcers within the last six months, use of anticoagulant therapy, thrombocytopenia)

• Chronic use of non-steroideal anti-inflammatory drugs or steroids

• Glomerular filtration rate less than 40 ml/min/1.73m2 according to MDRD formula [16]

• Presence of any life -threatening condition

• History of active substance or alcohol abuse within the last year or major psychiatric disorder

### Participating Centres

Eligible patients will be screened and recruited by two Diabetes outpatients services of Azienda USL di Modena (located in Modena and Sassuolo).

All of the exercise tolerance testing will be performed at Nuovo Ospedale Civile Sant'Agostino Estense - Medicina Cardiovascolare (Modena), results and ECG traces will be revised by three cardiologists (FT, PG and RM).

Coronary angiographies will be performed at local reference cardiac catheterization laboratory located in Nuovo Ospedale Civile Sant'Agostino Estense di Baggiovara by four expert cardiologists (ST, PM, CC and GD). This is a high volume procedure laboratory serving as the hub in our district.

### Participant enrolment

All eligible patients will be asked to participate. Once informed, they will be requested to sign a consent form. At the first visit they will be given a brochure containing detailed information about diabetes, hypertension, atherosclerosis and prevention. All participants will continue with regular follow ups scheduled by the referral diabetes outpatients service.

All smokers will be invited to quit. Physical activity and diet will be encouraged.

At the time of first visit a set of data will be collected:

Personal and clinical data regarding diabetes, cardiovascular risk factors, medications and blood pressure measurements will be collected. Information on laboratory parameters including lipid profile, serum creatinine, HbA1c will also be collected. Cardiovascular risk will be calculated according to the Italian risk chart provided by Istituto Superiore di Sanità [14].

All this data will be abstracted, reported in ad hoc forms and centrally collected.

During follow up the occurrence of any clinical event included in the end-points will be notified and verified through the registers of local Hospital admissions. After the completion of follow each patient will be contacted for the final visit.

### Randomization

Allocation to screening will be separately determined and communicated back by e-mail to diabetes services which will contact patients to schedule exercise testing a few days after their enrolment.

We chose a stratified block randomization with one to one allocation ratio. The allocation sequence was generated through a specific software available on the web [17], the scheme has been kept concealed.

Patients will be stratified according to age (≥ or < 60 years old); gender and cardiovascular risk score (≥ or < 20%).

### Pharmacological/Behavioural prevention of cardiovascular events

Pharmacological/behavioural prevention of cardiovascular events will be the same in both groups. Experimental and control group will continue to be followed at our local Diabetic Clinics that will try to provide maximal adherence the European Society of Cardiology Guideline [18] indications about control of hypertension, hypercholesterolemia and glycaemia.

### Screening procedures

Patients chosen for screening will be scheduled for exercise testing a few days after their enrolment.

A maximal symptom-limited exercise protocol will be used with a treadmill (*T*-*2100 Treadmill *- *GE Healthcare medical system*) according to the standard Bruce protocol. The test will be performed following the American Heart Association Guideline [10]. Twelve ECG leads will be recorded every minute and blood pressure will be measured manually at rest and every three minutes. Ventilatory oxygen consumption, expressed in multiples of resting requirements (METS), will be estimated by exercise duration.

The exercise test will be defined as maximal if the patient will reach 80% of the predicted exercise capacity according to Gulati formula [19]. Sub maximal tests without ECG signs and/or symptoms of ischemia will be considered not diagnostic and will not lead to any other procedure.

The exercise test will be considered positive if showing horizontal or downsloping ST segment depression of 1 mm or more calculated at 0.06-0.08 second after the J point at precordial leads (V3-V6).

Coronary angiography will be proposed to all patients with positive exercise testing.

Coronary angiography will be carried out within 30 days after the exercise testing. All angiography will be performed at our local reference cath-laboratory.

Coronary artery disease will be defined as being significant if lumen stenosis will be greater than 50% at level of left main or left anterior descending, or greater than 70% at level of circumflex or right coronary artery.

Treatment will be decided together with two consultant cardiologists after reviewing coronary anatomy. Treatment (surgery or stenting) will be determined keeping the European Guideline as a reference guide [18].

### Sample Size Estimation

The sample size estimates are based on the following assumptions:

• *Follow up for three years*

• *Event rate in screened population: *1.03/year

Based on existing data we can assume that about 80% patients will test negative [20] and run a risk of 0.97% per year [21] while the remaining 20% of patients will test positive running a risk of 1.03% per year after treatment according to the results of the ACIP trial [22]. This results in a mean risk of 1.03 per year within the group.

• *Event rate in follow up population: *2.6/year

The cardiac event rate reported in over 10000 Italian diabetic patients without evidence of cardiac disease was collected by the Italian Istituto Superiore di Sanità (Diabetes and Informatics Study Group): the cumulative incidence of cardiac events was 2.88% per year in men and 2.33% per year in women [23].

• *Two-sided tests of significance at an alpha = 0.05*

• *Power analysis of 0.80*

Given these assumptions, the total number of subjects to be enrolled is of 364 patients for each arm [24].

### Statistical analysis

The results will be evaluated based on the intention to treat analysis.

The incidence rates will be estimated using Kaplan-Meier survival curves that will be compared using logrank analysis. The treatment efficacy will be assessed by multivariate analyses using Cox's regression model.

### Interim Analyses

During the study interim analyses will be performed every year with the scope of verifying the correctness of the assumptions made for sample size estimation with regard to the primary end point event rate (this information can influence the duration of follow up) and to avoid unforecast excess of event rate in the treatment group.

### Economic evaluation

We will assess the incremental costs and the economic consequences of screening for asymptomatic coronary artery disease in diabetic patients with the aim of revascularization compared with usual care alone according to established methods for the analysis of patient-level data [25]. We will estimate all health care consumption and costs of both groups verifying hospital admissions from hospital records.

### Ethical consideration

The study will be conducted in accordance with European Commission guidelines for Good Clinical Practice and performed according to the revised Declaration of Helsinki. The study protocol was approved by local Ethical Committee.

The trial was registered at ClinicalTrial: the U.S. National Institute of Health registry of federally and privately supported clinical trials conducted in the United States and around the world [NCT00547872].

### Publication Policy

The study results will be submitted for publication in an appropriate journal irrespective of the outcome. Trial data will be reported according to the Consolidated Standards of Reporting Trials (CONSORT statements) [26]. The principal investigator will be responsible for timely generation of report manuscripts, and prior to submission the co-investigators will review and approve study results.

### Timing

September 2007 - Recruitment starts

December 2009 - Recruitment completed

December 2011 - Last participant completes 2-year follow-up

February 2012 - Analysis and publication of 2-year data

December 2012 - Last participant completes 3-year follow-up

February 2013 - Analysis of 3-year data and final publication

## Discussion

The study will be among the first randomised trials to investigate the effectiveness and cost-utility of a strategy of screening for unknown coronary artery disease in diabetic patients aimed at revascularisation. Recently the publication of DIAD trial results [27] demonstrates that screening asymptomatic diabetic patients by myocardial perfusion imaging without any treatment strategy does not affect outcome. This study cannot be considered the last word in this field because a screening plan should be performed only if treatment is available when positive cases are found. DIAD study design is not thought to test screening and treatment together but screening alone. Moreover the screening tool was myocardial perfusion imaging, a very sensitive and specific test, but with considerable radiation burden. In case of favourable results such a strategy would not be applicable to a large population due to costs and risks. Exercise tolerance testing is a simple and cheap test with low or no risk, it does not deliver radiations. In case of positive results the same strategy would be completely feasible in the *real world*.

Even the publication of BARI 2D [28] trial results does not represent the final answer to the dispute. BARI 2D was not designed as screening trial, patients with left main disease were excluded and patients with history of heart failure or ischemic heart disease were included. Furthermore a benefit in terms of major cardiovascular events was observed in patients undergone coronary artery bypass.

This study is designed as screening and treatment study and mandate coronary angiography for every patient with positive exercise testing. In case of coronary blockages, revascularization (percutaneous or surgical) is proposed. The results will help to establish if a screening strategy followed by revascularization is the best approach to high risk diabetic patients.

## Competing interests

The authors declare that they have no competing interests.

## Authors' contributions

FT conceived of the project, led the design and co-ordination of the trial and drafted the manuscript. FT and RM will be responsible for data collection.

RC, EP, SR and AB will be responsible for the recruitment and follow up of the patients enrolled. FT, PG and RM will perform the screening exercise testing and will be responsible for hospitalization for coronary angiography procedures.

ST, PM, CC and GT will perform coronary angiography FT, RM, PM, ST, GZ and BM are members of the steering committee, they will review data and they will be responsible for the publication of the trial.

## References

[B1] Fein F Scheur J Rifkin H, Porte D Jr Heart disease in diabetes mellitus: theory and practice 1990 New York: Elsevier 812 823

[B2] Bonow RO Bohannon N Hazzard W Risk stratification in coronary artery disease and special populations (review) Am J Med 1996 101 17S 22S 10.1016/S0002-9343(96)00312-9 8900333

[B3] Jouven X Lemaitre RN Rea TD Sotoodehnia N Empana JP Siscovick DS Diabetes, glucose level, and risk of sudden cardiac death Eur Heart J 2005 26 2142 2147 10.1093/eurheartj/ehi376 15980034

[B4] Haffner SM Lehto S Ronnemaa T Pyorala K Laakso M Mortality from coronary heart disease in subjects with type 2 diabetes and in nondiabetic subjects with and without prior myocardial infarction N Engl J Med 1998 339 229 234 10.1056/NEJM199807233390404 9673301

[B5] Greenland P Lloyd-Jones D Definign a rational approach to screening for cardiovascular risk in asymptomatic patients J Am Coll Card 2008 52 330 332 10.1016/j.jacc.2008.04.029 18652939

[B6] Bax JJ Inzucchi SE Bonow RO Schiuf JD Freeman MR Barrett EJ Cardiac Imaging for risk stratification in diabetes Diabetes Care 2007 30 1295 1304 10.2337/dc06-2094 17259467

[B7] Beller GA Noninvasive screening for coronary atheroscelrosis and silent ischemia in asymptomatic type 2 diabetic patients: is it appropriate and cost-effective? J Am Coll Card 2007 49 1918 1923 10.1016/j.jacc.2007.01.079 17498575

[B8] Grimes DA Schulz KF Uses and abuses of screening tests Lancet 2002 359 881 884 10.1016/S0140-6736(02)07948-5 11897304

[B9] Ashley EA Myers J Froelicher V Exercise testing in clinical medicine Lancet 2000 356 1592 1597 10.1016/S0140-6736(00)03138-X 11075788

[B10] Gibbons RJ Balady GJ Bricker JT Chaitman BR Fletcher GF Froelicher VF Mark DB McCallister BD Mooss AN O'Reilly MG Winters WL Jr ACC/AHA 2002 guideline update for exercise testing: a report of the American College of Cardiology/American Heart Association Task Force on Practice Guidelines (Committee on Exercise Testing) American College of Cardiology 2002 http://www.acc.org/qualityandscience/clinical/guidelines/exercise/dirindex.htm

[B11] Barett EJ Ginsberg HN Pauker SG Rutherford JD Smith SC Young LH Zimmerman BR Consensus development conference on the diagnosis of coronary heart disease in people with diabetes Diabetes Care 1998 21 1551 1559 10.2337/diacare.21.9.1551 9727908

[B12] Fowler-Brown A Pignone M Pletcher M Tice JA Sutton SF Lohr KN Exercise Tolerance Testing To Screen for Coronary Heart Disease: A Systematic Review for the Technical Support for the U.S. Preventive Services Task Force Ann Intern Med 2004 140 W9 W24 15069009 10.7326/0003-4819-140-7-200404060-w1

[B13] Mehta SR Eikelboom JW Demers C Maggioni AP Commerford PJ Yusuf S Congestive heart failure complicating non-ST segment elevation acute coronary syndrome: incidence, predictors, and clinical outcomes Can J Physiol Pharm 2005 83 98 103 10.1139/y05-003 15759056

[B14] Il Progetto Cuore http://www.cuore.iss.it/sopra/calc-rischio.asp

[B15] Calmieri L Vanuzzo D Panico S Ferrario M Giampaoli S a nome del Gruppo di Ricerca del Progetto CUORE - Studi Longitudinali La strategia dell'alto rischio. Il rischio di primo evento cardiovascolare maggiore negli uomini Ital Heart J 2004 5 54s 58s 15615363

[B16] Levey AS Greene T Kusek JW Beck GL MDRD Study Group A simplified equation to predict glomerular filtration rate from serum creatinine [abstract] J Am Soc Nephrol 2000 11 155A

[B17] Randomization.com http://www.randomization.com

[B18] The task force on Diabetes and Cardiovascular Diseases of European Society of Cardiology (ESC) and of the European Association for the Study of Diabetes (EASD) Guidelines on diabetes, pre-diabetes, and cardiovascular diseases Eur Heart J 2007 28 88 136 10.1093/eurheartj/ehl260 17220161

[B19] Gulati M Black HR Shaw LJ Arnsdorf MF Merz NB Lauer MS Marwick TH Pandey DK Wicklund RH Thisted RA The prognostic value for exercise capacity in woman N Engl J Med 2005 353 468 475 10.1056/NEJMoa044154 16079370

[B20] Bacci S Villella M Villella A Langialonga T Grilli M Rauseo A Mastroianno S De Cosmo S Fanelli R Trischitta V Screening for silent myocardial ischemia in type 2 diabetic patients with additional atherogenic risk factors: applicability and accuracy of the exercise stress test Eur J Endocrinol 2002 147 649 654 10.1530/eje.0.1470649 12444897

[B21] Faglia E Favales F Calia P Paleari F Segalini G Gamba PL Rocca A Musacchio N Mastropasqua A Testori G Rampini P Moratti F Braga A Morabito A Cardiac Events in 735 type 2 diabetic patients who underwent screening for unknown coronary heart disease Diabetes Care 2002 25 2032 2036 10.2337/diacare.25.11.2032 12401752

[B22] Bourassa MG Knatterud GL Pepine CJ Sopko G Rogers WJ Geller NL Dyrda I Forman SA Chaitman BR Sharaf B Davies RF Conti R for the ACIP Investigators Asymptomatic Cardiac Ischemia Pilot (ACIP) Study - Improvement of Cardiac Ischemia at 1 year after PTCA and CABG Circulation 1995 92 1 7 7586390 10.1161/01.cir.92.9.1

[B23] Avogaro A Giorda C Maggini M Mannucci E Raschetti R Lombardo F Spila-Alegian S Turco S Velassi M Ferrannini E for the Diabetes and Informatics Study Group, Association of Clinical Diabetologists, Istituto Superiore di Sanità Incidence of Coronary Heart Disease in Type 2 Diabetic Men and Women Diabetes Care 2007 30 1241 1247 10.2337/dc06-2558 17290034

[B24] Rosner B Hypothesis testing - Estimation of Sample Size and Power for Comparing Two Means Fundamentals of Biostatistics 2006 Sixth Cengage Learning 416 426

[B25] Drummond MF Sculpher MJ Torrance GW O'Brien BJ Stoddart GL Methods for the economic evaluation of health care programmes 2005 Third New York, USA: Oxford University Press

[B26] CONSORT - Trasparent Reporting of Trials http://www.consort-statement.org

[B27] Young LH Wackers FJT Chyun DA Davey JA Barret EJ Taillefer R Heller GV Iskandrian AE Wittlin SD Filipchuk N Ratner RE Inzucchi SE Cardiac Outcomes after screening for asymptomatic coronary artery disease in patients with type 2 diabetes JAMA 2009 301 1547 1555 10.1001/jama.2009.476 19366774 PMC2895332

[B28] The BARI 2D Study Group A randomized trial of therapies for type 2 diabetes and coronary artery disease N Engl J Med 2009 360 2503 2515 10.1056/NEJMoa0805796 19502645 PMC2863990

